# NGS-Panel Diagnosis Developed for the Differential Diagnosis of Idiopathic Toe Walking and Its Application for the Investigation of Possible Genetic Causes for the Gait Anomaly

**DOI:** 10.1055/s-0043-57230

**Published:** 2023-04-21

**Authors:** David Pomarino, Anna Emelina, Jens Heidrich, Kevin Rostásy, Svenja Schirmer, Jan O. Schönfeldt, Anneke Thren, Ferdinand Wagner, Johanna Ronja Thren, Nina Berger

**Affiliations:** 1 Praxis Pomarino, Hamburg, Germany; 2 Labor Dr. Heidrich und Kollegen MVZ GmbH, Hamburg, Germany; 3 Department of Orthopedics and Trauma Surgery, Musculoskeletal University Center Munich, University Hospital, Ludwig-Maximilians-University, Munich, Germany; 4 Anthropology Department, Durham University, Durham, United Kingdom; 5 Kinderklinik Datteln, Datteln, Germany; 6 SANA Klinikum Offenbach, Offenbach, Germany; 7 Institut für Kinderneurologie, Hamburg, Germany; 8 Kinderorthopädie am Pferdeturm, Hanover, Germany

**Keywords:** idiopathic toe walking, pes cavus, toe walking family history, cause toe walking, ITW

## Abstract

Idiopathic toe walking (ITW) describes a condition affecting approximately 4.5% of children. Toe walking is an accompanying symptom for many hereditary disorders. This retrospective study uses next-generation sequencing-panel-diagnosis to investigate the feasibility of genetic testing to research the possible genetic causes of ITW and for differential diagnosis.

Data were taken from our inhouse database, the minimum age for participants was 3 years. Underlying neurological or orthopaedic conditions were tested for and ruled out prior to diagnosing ITW. Patients, who experienced complications before, during or immediately after birth, children with autism, and patients toe walking less than 50% of the time were excluded.

Eighty-nine patients were included in the study, in which 66 (74.2%) patients were boys and 23 (25.8%) girls. Mean age at testing was 7.7 years (range: 3–17 years). Fifteen of the 89 patients included in the study (16.9%) had a genetic variant identified as likely pathogenic or pathogenic by the genetics laboratory. Additionally, we found 129 variants of uncertain significance. About 65.2% of patients showed a pes cavus foot deformity, 27% of patients reportedly had at least one relative who also displayed the gait anomaly, and 37.1% had problems with their speech development.

Despite the limitations of the sample size and the scope of our genetic testing targets, our results indicate that research into the genetic causes of ITW could better our understanding of the causes of ITW in otherwise healthy children, to help develop novel methods to detect serious conditions early. ITW could be an early onset symptom for further hereditary conditions.

## Introduction

The following is an exploratory retrospective study into possible strategies to investigate the hypothesis that there is no link between idiopathic toe walking (ITW) and several genetic variations in a gene panel linked to functions that have the potential to affect gait. Before we present the study, we will provide a background discussion on the term ITW and provide a rationale for the merits of investigating whether the gait anomaly could have a genetic cause.

The term habitual toe walking or idiopathic toe walking is usually used as a diagnosis of exclusion for healthy children who persist in walking on their toes after they pass the age when they should normally have transitioned to a heel-toe gait. 1

ITW was first described by Hall et al in 1967, they used the term “congenital short tendon calcaneus,” by describing the anomaly as “congenital” they were already implying a possibly hereditary cause for the condition. 2 Their diagnosis referred to otherwise healthy children who were displaying a toe walking gait in the absence of a known neurological or orthopaedic cause. Since then, various causal hypotheses and possible contributing factors have been put forward, such as a possible underlying disorder of the sensory nervous system, 3 4 problems with isolated aspects of motor development, 5 or the excessive use of so-called “baby walkers.” 6 Finally, ITW has also been described as “habitual toe walking,” which suggests a voluntary choice of walking pattern by the patient as the cause of the condition. To date, the etiology of ITW remains unclear, despite the prevalence of the condition among young people. A large study of 1,401 healthy Swedish children at the age of approximately 5.5 years found that approximately 4.5% of those children displayed ITW. 7

When research on the potential causes of toe walking began, researchers were quick to suspect underlying genetic disorders with autosomal inheritance, variable gene expression, and heterogeneous etiology as a reason for the anomaly. 8 9 Levine described toe walking in five members of a family with short tendons as the sole finding and noted that this condition caused some of those family members to walk on their toes, but that they were otherwise healthy. 8 He concluded that the condition affecting this family could be dominantly inherited. Similarly, Katz and Mubarak presented a case of dominantly inherited tendon Achilles contracture in a patient leading to a minor impairment of gait. 9

The theory of a hereditary cause for ITW is further supported by reports of a relatively high prevalence of idiopathic toe walkers, who have at least one other relative who currently is a toe walker or who had in the past displayed the gait anomaly. Engström and Tedroff reported that 42 of 26 active idiopathic toe walkers and 38% of 37 inactive idiopathic toe walkers (who had seized toe walking before the age of 5.5 years) had a first- or second-degree relative who had also been a toe walker. 10 Other studies have reported a positive family history in 30 to 71% of patients. 11 12 13 14 15

Multiple hereditary disorders are known to be associated with developmental delay. There is evidence that concomitant developmental disorders or delays, affecting language, speech, or motor development, are more frequent in ITW children. Engström and Tedroff stated that “children with ITW as a group ( *n* = 51) displayed more neuropsychiatric problems than a normative group of age matched children ( *n* = 385),” though significance was only reached in the items “Gross Motor Function” and “General Learning” in the age group 9 to 12 years. 10 The same authors reported in 2018 that 10% out of 70 toe walking children had concomitant neurodevelopmental problems such as autism spectrum disorder, unspecific delayed development, or attention deficit/hyperactivity disorder. 7 Other groups noted a delay in language development in 27 to 33% of their toe walking population, fine motor development, visuomotor or gross motor development. 12 16 Furthermore, studies by Barrow et al and Soto-Insuga et al found 20 to 21% of their respective autism spectrum disorder and attention-deficit hyperactivity disorder population demonstrated an accompanying persistent toe walking gait. 17 18

There are also histopathological findings in some ITW patients suggesting underlying neuromuscular pathologies. Eastwood et al completed a detailed examination of the calf muscle fibers of 24 idiopathic toe walkers and discovered atrophic, angular fibers, suggesting underlying neuropathic activities in 42% of patients. 19 Other studies focused on sensory and motor function, reporting poorer average function in idiopathic toe walkers. 5 20 21 22

There are many hereditary pathologies that can cause a toe walking gait alongside other symptoms. The main group of diseases whose symptoms include toe walking are neuromuscular diseases such as peripheral neuropathies (Charcot-Marie-Tooth disease), congenital muscular dystrophies, and progressive muscular dystrophies. 23

Charcot-Marie-Tooth disease is the most common form of an inherited polyneuropathy. 24 The disease is transmitted by both autosomal dominant and autosomal recessive inheritance. 25 26 In 2018, Haynes et al found in a retrospective study that 30% of 74 genetically tested toe-walkers had identifiable gene mutations, five of them had Charcot-Marie-Tooth disease type 1A, one had Angelman syndrome, one had Sjogren-Larsson syndrome, one had x-linked syndromic mental retardation-14, and one had multiple synostoses syndrome. 27 While these children did not have ITW, but toe walking linked to a known condition, the multitude of neurological and developmental disorders for which toe walking is a symptom means that routine genetic testing of toe walking with an unknown cause could help detect previously undetected conditions early. Engström and Tedroff noted in a 2012 study that, “for children with a neuropsychiatric diagnosis or developmental delay, the total prevalence for active or inactive toe-walking was 7 (41.2%) of 17.” 10 For this reason, many parents are concerned that persistent toe walking in their children could be a sign of an undetected serious condition—genetic testing has the potential of minimizing this worry.

Another pathology which has been linked to toe walking is Ullrich congenital muscular dystrophy. Mutations in the collagen VI genes COL6A2 and COL6A3 are associated with the development of either the severe phenotype Ullrich dystrophy or the mild-to-moderate phenotype of a Bethlem myopathy. 28 Myotonia congenita, contributing 75% of nondystrophic myotonia, is caused by mutations in the CLCN1 gene. 28

To our knowledge, there are currently no studies examining the potential genetic causes of toe walking in ITW patients (we searched the following databases: kofam.ch; basg.av.at; drks.de; clinicaltrialsregister.eu; clinicaltrials.gov).

By performing this study, we want to address the much-discussed possibility of genetic variants as a possible cause for the toe walking gait. We do this to investigate the merits of further, more in-depth research to investigate specific genetic variants that could be linked to toe walking and to describe a gene panel that could be used to find any underlying pathologies linked to known genetic conditions early on. Since toe walking is an important early symptom for a wide and growing range of motor developmental and neurological disorders, we also hope to contribute to the development of strategies to diagnose and treat such conditions from a young age. For the purposes of this study, we put up the null hypothesis that variants in one or several of 49 distinct genes related to myopathies and neuropathies, tested using a commercially available next-generation sequencing (NGS) panel, cannot be brought in relation with the symptom “toe walking gait” in any of the patients diagnosed with “idiopathic toe walking.” The aim of our study is to confirm or rebut this hypothesis. Secondary endpoints are the detection of possible areas for more complex future research into the possible significance of future research projects and to postulate an improved diagnostic process for the possible causes of toe walking in children with no clear indication for an underlying neurological or orthopaedic cause.

## Materials and Methods

We searched our inhouse database in our specialist practice for gait anomalies for patients that have been diagnosed as “idiopathic toe walkers” by their assigned pediatrician. The minimum age at the time of diagnosis for inclusion in this retrospective study was 3 years. Underlying acquired or congenital pathologies such as cerebral palsy or tethered cord syndrome were routinely tested for and ruled out prior to the diagnosis of ITW being formulated in the specialist practice for gait anomalies. The retrospective study excluded patients from the database, who experienced complications before, during or immediately after their birth. Additionally, where an alteration of the monosynaptic reflexes, pronounced muscular weakness in the triceps surae muscle, or an asymmetry was detected during the initial clinical examination, patients were recommended for further neurological assessment. If this additional examination and testing resulted in the diagnosis of a neurological disorder, the relevant patients were excluded from this study. No children with autism were included in the study. Lastly, patients who displayed severe orthopaedic deformities, that is, limb length discrepancies, axis deformities, scoliosis or severe foot deformities, were also excluded.

Patients suitable for inclusion had a reported toe walking gait (based on the time spent walking barefoot or in socks; as estimated by the caregivers) during at least 50% of the time at their initial visit, as reported by their parent of guardian and observed by the examiner.

Only patients who had undergone a genetic test using a specific NGS panel comprising 49 genes intended to detect any hereditary neuro- and myopathies with a potential link to toe walking were eligible for inclusion. Children who had received other forms of genetic testing such as exome sequencing were not selected for inclusion.

**Fig. 1:**
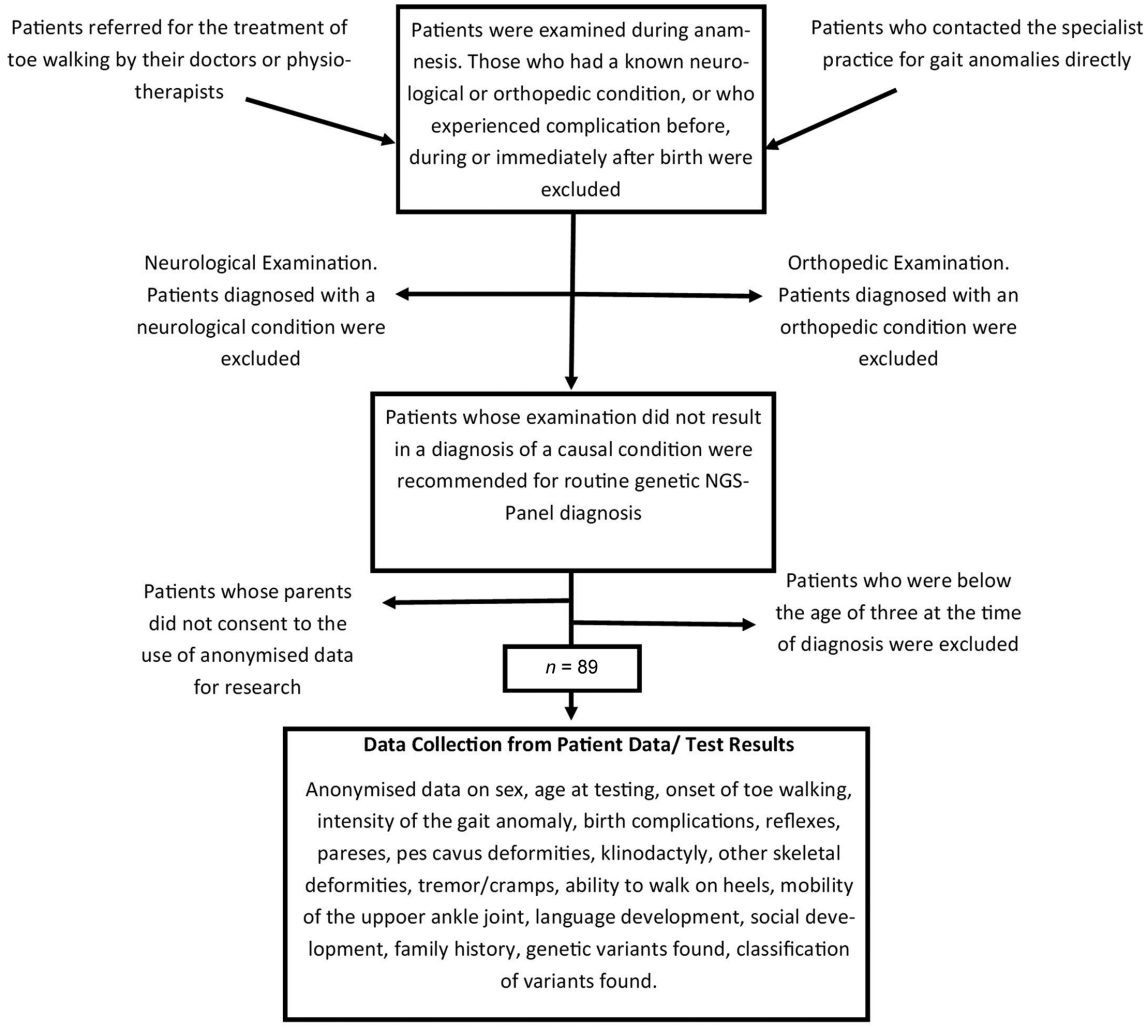
Retrospective recruitment process. NGS, next-generation sequencing.

The genetic testing for any patients proposed for inclusion was routinely recommended as part of their diagnostic process, and testing was facilitated using saliva samples, collected by the individual patient's treating (neuro)pediatrician as part of the routine diagnostic process. Written consent for the use of the results for the purpose of scientific research was routinely obtained prior to testing and prior to inclusion of the data in the database for all patients who were eligible for inclusion in the proposed study. All data used for research was formally anonymized. Explanations of the results of the laboratory analysis and any important implications of the test results were routinely provided. The study was approved by the ethical board of LMU Clinic in Munich, the project number is 22–0812. Fig. 1 .

The NGS panel that was used to test the hypothesis has been developed by a German laboratory, using the HPO database ( https://hpo.jax.org/app/ ). It comprises 49 genes that can be linked to several relevant neuro- and myopathies. Genes exclusively associated with severe forms of neuro- or myopathies leading to intrauterine fetal death or intrauterine onset of severe neurological symptoms are not included in the panel.

The NGS panel is an Amplicon-based Customer Panel for the Ion Torrent Platform (Thermo Fisher Scientific Headquarters, Waltham, Massachusetts, United States of America) for genes that are clinically relevant for neuropathies. Sequencing of the coded exons is inclusive of the exon-intron boundaries. Intronic regions are scored up to ± 10 base pairs from the exon boundary.

The panel analyzes the following genes: AIFM1 (NM_004208.3, MIM*300169), ALS2 (NM_020919.3, MIM*606352), ATM (LRG_135t1, MIM*601556), ATXN1 (LRG_863t1, MIM*601556), ATXN2 (NM_002973.3, MIM*601517), ATXN3 (NM_004993.5, MIM*607640), ATXN7 (NM_000333.3, MIM*607640), CACNA1A (LRG_7t1, MIM*601011), CAV3 (LRG_329t1, MIM*601253), CHRNE (LRG_1254t1, MIM*100725), CLCN1 (NM_000083.2, MIM*118425), COL6A2 (LRG_476t1, MIM* 120240), COL6A3 (LRG_473t1, MIM*120250), CREBBP (NM_004380.3, MIM*600140), DHTKD1 (NM_018706.6, MIM*614984), EGR2 (LRG_234t1, MIM*129010), EPHB4 (NM_004444.4, MIM* 600011), FBLN5 (LRG_240t1, MIM*611104), FGD4 (LRG_240t1, MIM*611104), FXN (NM_000144.4, MIM*606829), GARS1 (NM_002047.3, MIM*600287), GDAP1 (LRG_244t1, MIM*606598), IQSEC2 (NM_001111125.2, MIM*300522), KCNC3 (NM_004977.2, MIM*176264), KMT2C (NM_170606.2, MIM”606833), LITAF (LRG_253t1, MIM*603795), MED25 (LRG_368t1, MIM*6101970), MFN2 (LRG_255t1, MIM*608507), MORC2 (NM_001303256.2, MIM*616661), MPZ (LRG_256t1, MIM* 159440), NAGLU (NM_000263.3, MIM*609701), NDRG1 (LRG_258t1, MIM*605262), NEFL (LRG_259t1, MIM*162280), OPA1 (LRG_337t2, MIM*605290), PMP22 (LRG_263t1, MIM*601097), POLG (LRG_765t1, MIM*174763), PRX (LRG_265t2,*605725), PYGM (NM_005609.3, MIM*608455), RETREG1 (LRG_363t1, MIM*613114), SATB2 (NM_015265.3, MIM*608148), SBF1 (NM_002972.3, MIM*603560), SBF2 (LRG_267t1, MIM*607697), SH3TC2 (LRG_371t1, *605713), TRPV4 (LRG_372t1, MIM*605427), TTN (LRG_39111 It2, MIM*188840), TTR (LRG_416t1, MIM*176300), TRIO (NM_007118.3, MIM*601893), ZFYVE26 (NM_015346.3, MIM*612012).

Data are analyzed using the SegNext module and using the SeqPilot software (version 5.0.0 build 508). The raw data has a sequence depth (coverage) of at least 30-fold and variants with an allele read frequency of more than 20%.

Genetic variants were divided into five categories according to guidelines by Greenblatt et al. Hum Mutat 29:1282ff: category 1 (benign), category 2 (likely benign), category 3 (variant with unclear significance), category 4 (likely pathogenic), and category 5 (pathogenic). 29 Variants that can be assigned to categories 1 and 2 according to this classification were excluded during the process of bioinformatic analysis and are not usually reported in the findings. However, they can be specified upon request. If required, in silico analyses were performed, for example, using the programs Mutation Taster, PolyPhen2, and/or Mutation Assessor. The following databases were used: HGMD professional (The Human Gene Mutation Database ( https://portal.biobase-international.com/hgmd/pr o/gene.ph p?), LOVD -IARC (Leiden Open Variation Database, http://grenada.lume.ni/LSDB_list/Isdbs ), dbSNP, ClinVar, as well as, when and as required, more specialized databases. Variants outside the analyzed areas in the examined genes (for example in untranslated, regulatory gene areas), in regions with multiple copies of high sequence homology, repeat variants as well as copy number variants of single exons or when a complete gene cannot be detected and thus cannot be excluded. In addition, mosaics with a low frequency component cannot be excluded with certainty. Although unlikely, it is also possible that new scientific knowledge could change the assessment of the pathogenicity of variants at a later stage. The sensitivity for detection of clinically relevant variants for the applied methods is more than 96%.

All anamnestic parameters of the toe walking patients were routinely recorded using a standardized questionnaire. The parameters covered by the questionnaire include a perinatal anamnesis, questions regarding the timing of milestones of motor development, the onset and severity of toe walking, the occurrence of toe walking as an approximate percentage of time per day, the social and speech development of the child according to parental reports, their stamina and whether there was a higher propensity for toe walking when the child seemed agitated, nervous, excited, etc. The questionnaire also includes sections to record the results of the physical examination, including details on any foot deformities and the mobility of the upper ankle joint. All patients who would be included in the proposed study were examined using the standard process. Dorsiflexion and plantarflexion of the upper ankle joint were tested in a straight knee and a 90-degree bent knee position and measured using a hand-held goniometer. The following clinical signs that would have arisen during the physical examination were also eligible for inclusion in the database: tremor, cavus deformity of the foot, reflex anomalies.

## Results

Eighty-nine patients were included in the study. There were 66 (74.2%) boys and 23 (25.8%) girls. Mean age at testing was 7.7 years (range: 3–17 years). Forty-four patients (49.4%) had been walking on their toes since they started walking. Twelve (13.5%) patients developed a toe walking a few months after beginning to walk, 18 (20.2%) started toe walking between the ages of 3 and 6, 7 (7.9%) patients were aged between 7 and 10 when they began walking on their toes, and one (1.1%) patient started walking on their toes at a later age, namely after the age of 10. The onset of toe walking was unknown in eight patients (9%).

All patients had a history of seeing different doctors before presenting for treatment at our clinic. Mean dorsiflexion of the foot measured in a straight knee position was 5 degrees (range: −10–10 degrees) and 8.5 degrees in the bent knee position (range: −10–20 degrees).

Twenty-four patients (27%) had a positive family history for toe walking, where at least one close relative was also reported to have the gait anomaly. Fifty-eight (65.2%) patients showed a pes cavus deformity of the foot, three (3.4%) exhibited a funnel chest, and 5 (5.6%) displayed a tremor during the initial clinical examination for toe walkers. None of the children showed an indication for a mental developmental disorder. Thirty-three (37.1%) patients had problems with their motor abilities when speaking, causing mild speech impairments as observed by the parents and reported by the examiner.

Of the 89 patients who were eligible for genetic testing using the genetic panel comprising of 49 genes, 88 patients (98.9%) had at least one genetic variant in the 49 genes that were tested. Fifteen of the 89 patients included in the study (16.9%) were found to have at least one genetic variant characterized as likely pathogenic or pathogenic; this group of patients had an additional 17 variants of uncertain significance ( Tables 1 and 2 ).

**Table 1:** Clinical symptoms found in the study population

Patient group	Cavus deformity of the foot	Family history	Speech problems	Tremor
Total study population *n* = 89	58 (65.5%)	24 (27%)	33 (37.1%)	5 (5.6%)
Patients with min. one variant of uncertain significance *n* = 69	45 (65.2%)	20 (29%)	25 (36.2%)	3 (4.3%)
Patients with min. one likely pathogenic/pathogenic variant, *n* = 15	7 (46.7%)	2 (13.3%)	2 (13.3%)	1 (6.7%)

**Table 2:** Pathogenic and likely pathogenic variants found in the study population

Genetic variants by patient	Pathogenicity	Conditions linked to the gene
ATM c.8545C > T	Pathogenic	Ataxia-telangiectasia; mantle cell lymphoma; ataxia-telangiectasia; breast cancer
CACNA1A c.3787G > A	Likely pathogenic	Familial paroxysmal ataxia; familial or sporadic hemiplegic migraine; epileptic encephalopathy, early infantile; benign paroxysmal torticollis of infancy; migraine, familial hemiplegic; alternating hemiplegia of childhood; episodic ataxia, type 2; spinocerebellar ataxia 6; spinocerebellar ataxia type 6; Lennox-Gastaut syndrome; non-specific early-onset epileptic encephalopathy
CHRNE c.346A > G	Likely pathogenic	Postsynaptic congenital myasthenic syndromes; myasthenic syndrome, congenital, 4c, associated with acetylcholine receptor deficiency; myasthenic syndrome, congenital, 4a, slow-channel; myasthenic syndrome, congenital, 4b, fast-channel
CHRNE c.488C > T	Likely pathogenic	Postsynaptic congenital myasthenic syndromes; myasthenic syndrome, congenital, 4c, associated with acetylcholine receptor deficiency; myasthenic syndrome, congenital, 4a, slow-channel; myasthenic syndrome, congenital, 4b, fast-channel
COL6A3 c.7174G > A OPA1 c.113_130del,	Pathogenic Pathogenic	Congenital muscular dystrophy, Ullrich type; dystonia 27; primary dystonia, dyt27 type; Bethlem myopathy; Bethlem myopathy 1; Ullrich congenital muscular dystrophy 1 Autosomal dominant optic atrophy plus syndrome; autosomal dominant optic atrophy, classic form; optic atrophy with or without deafness, ophthalmoplegia, myopathy, ataxia, and neuropathy; mitochondrial DNA depletion syndrome 14 (encephalo-cardiomyopathic Type); Behr syndrome; optic atrophy 1
PMP22 c.353C > T	Likely pathogenic	Roussy-Levy syndrome; Roussy-Levy hereditary areflexic dystasia; neuropathy, inflammatory demyelinating. OMIM:118300; Charcot-Marie-Tooth disease and deafness; hypertrophic neuropathy of Dejerine-Sottas; acute inflammatory demyelinating polyradiculoneuropathy; hereditary neuropathy with liability to pressure palsies; neuropathy, hereditary, with liability to pressure palsies; charcot-marie-tooth disease type 1a
PMP22 Complete Deletion of Exon 2	Pathogenic	Roussy-Levy syndrome; Roussy-Levy hereditary areflexic dystasia; neuropathy, inflammatory demyelinating; Charcot-Marie-Tooth disease and deafness; hypertrophic neuropathy of Dejerine-Sottas; acute inflammatory demyelinating polyradiculoneuropathy; hereditary neuropathy with liability to pressure palsies; neuropathy, hereditary, with liability to pressure palsies; Charcot-Marie-Tooth disease type 1a
PMP22 Gene Duplication	Pathogenic	Roussy-Levy syndrome; Roussy-Levy hereditary areflexic dystasia; neuropathy, inflammatory demyelinating; Charcot-Marie-Tooth disease and deafness; hypertrophic neuropathy of Dejerine-Sottas; acute inflammatory demyelinating polyradiculoneuropathy; hereditary neuropathy with liability to pressure palsies; neuropathy, hereditary, with liability to pressure palsies; Charcot-Marie-Tooth disease type 1a
POLG c.752C > T, POLG c.1760C > T	Pathogenic Pathogenic	Progressive external ophthalmoplegia with mitochondrial DNA deletions, autosomal dominant 1; autosomal dominant progressive external ophthalmoplegia; recessive mitochondrial ataxia syndrome; progressive external ophthalmoplegia with mitochondrial DNA deletions, autosomal recessive; Alpers-Huttenlocher syndrome; mitochondrial DNA depletion syndrome 4a (Alpers type); sensory ataxic neuropathy-dysarthria-ophthalmoparesis syndrome; mitochondrial neurogastrointestinal encephalomyopathy; mitochondrial DNA depletion syndrome 1; autosomal recessive progressive external ophthalmoplegia; mitochondrial DNA depletion syndrome 4b; sensory ataxic neuropathy, dysarthria, and ophthalmoparesis
POLG c.926G > A SATB2 c.1880G > T	Pathogenic Likely pathogenic	SATB2-associated syndrome due to A chromosomal rearrangement progressive external ophthalmoplegia with mitochondrial DNA deletions, autosomal dominant 1; autosomal dominant progressive external ophthalmoplegia; recessive mitochondrial ataxia syndrome; progressive external ophthalmoplegia with mitochondrial DNA deletions, autosomal recessive; Alpers-Huttenlocher syndrome; mitochondrial DNA depletion syndrome 4a (Alpers type); sensory ataxic neuropathy-dysarthria-ophthalmoparesis syndrome; mitochondrial neurogastrointestinal encephalomyopathy; mitochondrial DNA depletion syndrome 1 (MNGIE type); autosomal recessive progressive external ophthalmoplegia; mitochondrial DNA depletion syndrome 4b; sensory ataxic neuropathy, dysarthria, and ophthalmoparesis satb2-associated syndrome due to a chromosomal rearrangement
PYGM c.2056G > A	Pathogenic	Glycogen storage disease V; glycogen storage disease due to muscle glycogen phosphorylase deficiency
PYGM c.660G > A	Likely pathogenic	Glycogen storage disease V; glycogen storage disease due to muscle glycogen phosphorylase deficiency
SH3TC2 c.1402_1403delinsT	Likely pathogenic	Charcot-Marie-Tooth disease, type 4c; mononeuropathy of the median nerve, mild; Charcot-Marie-Tooth disease type 4c
TTN c.16063G > A	Likely pathogenic	Myopathy, myofibrillar, 9, with early respiratory failure; autosomal recessive centronuclear myopathy; Salih myopathy; tibial muscular dystrophy, tardive; tibial muscular dystrophy; familial isolated dilated cardiomyopathy; hereditary myopathy with early respiratory failure; classic multiminicore myopathy; muscular dystrophy, limb-girdle, autosomal recessive 10; cardiomyopathy, familial hypertrophic, 9; cardiomyopathy, dilated, 1 g
TTN c.33742 + 1G > T	Pathogenic	Myopathy, myofibrillar, 9, with early respiratory failure; autosomal recessive centronuclear myopathy; Salih myopathy; tibial muscular dystrophy, tardive; tibial muscular dystrophy; familial isolated dilated cardiomyopathy; hereditary myopathy with early respiratory failure; classic multiminicore myopathy; muscular dystrophy, limb-girdle, autosomal recessive 10; cardiomyopathy, familial hypertrophic, 9; cardiomyopathy, dilated, 1 g

Descriptions of the genetic conditions currently known to be associated with variants in the respective genes were compiled after consulting the omim.org database.

The interpretations were based on the available knowledge on the variants to date and the results of the clinical examination of each individual patient, as interpreted by the laboratory.

## Discussion

To our knowledge, this retrospective study provides the only large-scale exploratory genetic analysis whether there could be a link between ITW and genetic variants previously not known to be associated with the conditions. The purpose of this study was to assess the potential usefulness of future research in this area, which is why we present our results despite the limitations of the sample size and the scope of genetic testing targets. By performing this study, we found pathogenic or likely pathogenic variants in 17% of our patients in one or several of 49 distinct genes related to myopathies and neuropathies that could be brought in relation with the symptom “toe walking gait” and we therefore reject our null hypothesis.

Secondary endpoints are the detection of possible areas for more complex future research into the possible significance of future research projects and to postulate an improved diagnostic process for the possible causes of toe walking in children with no clear indication for an underlying neurological or orthopaedic cause. The presence of such variants was interpreted as making a genetic cause for the gait anomaly more likely. It was explicitly not interpreted as meaning that the patient has the condition the variant is associated with. Furthermore, it does not indicate any specific mechanism through which the variant could contribute to the gait anomaly. Many of the variants and conditions are characterized by heterogeneity of symptoms in carriers. Consequently, the results reported indicate initial findings and demonstrate the use of the diagnostic method. We found an additional 129 variants of uncertain significance in a total of 69 patients, and we cannot exclude the possibility that some of these additional variants could have contributed to the gait anomaly. Additionally, we found that 65.2% of patients showed a pes cavus deformity and 27% of patients reportedly had at least one close relative who also displayed the gait anomaly, while 37.1% had problems with their speech development. We have considered these combined findings and reject our hypothesis that there is no indication of a link between ITW and an underlying genetic cause. We conclude that the result shows that further research into the genetic causes of toe walking is justified—not least because such research could provide valuable insight into the diagnosis of more serious diseases. There are some important arguments for the use of genetic diagnostic methods outside the study results we have listed above. Parents of children with ITW often worry that there could be a risk of a serious undetected underlying condition and the treatment and interpretation of ITW are inconsistent. Parents often seek out multiple options for diagnosis and treatment. An option for routine genetic diagnosis which employs the diagnostic panel can offer them a pathway that can provide increased clarity about the risk that the gait anomaly their child is displaying could be associated with undetected underlying conditions.

In case of finding a genetic variant with a suspected pathological impact, treatment can be tailored to the individual patient. Our previous research has shown that toe walking can be a previously unknown initial symptom of a known genetic conditions. We have to date published two case studies where we were able to diagnose patients who had visited our practice for gait anomalies at a young age presenting with no pronounced symptoms of a neurological or orthopaedic condition; their main symptom at the time was toe walking. By using the NGS-panel, prior to this study, we were able to identify an undetected spinocerebellar ataxia 13 in a boy and a Menke-Hennekam syndrome in a girl. 30 31 Following both diagnoses, the gait anomaly could be treated alongside a recommendation for treatment of the conditions and a clearer prognosis for the children's future development. In this way, the further genetic testing could be used for the generation of knowledge and as a novel method to detect progressive genetic conditions, with the potential for more research to generate further insights.

Currently, many pediatricians do not pay particular attention to toe walking, often considering it a temporary phenomenon. However, in persistent cases, the gait anomaly may often require treatment to prevent progressive deformities and pain in later life, even for children who are otherwise healthy. Furthermore, doctors should be wary of toe walking as a possible early indicator of the presence of more serious hereditary diseases. The use of the genetic testing helps parents and doctors have a greater degree of clarity on the disease risks and possible resulting treatment risks. Often testing provides reassurance and avoids overtreatment and mounting costs to families and/or the health system as a result of attempts to establish the likelihood of a child having a previously undetected hereditary condition linked to toe walking.

There are several limitations to the conclusions we can draw based on the data we have discussed in this study. The sample size was limited given the large number of genes included in the panel and the breadth of conditions variants could be associated with. Second, due to the limited data available to date, we have little clarity on if and, if yes, how each individual variant could be linked to the clinical symptoms of toe walking. Our genetic testing targets were formulated very broadly, which means our results should be primarily interpreted as an encouraging indicator for future research on a larger scale or in-depth discussions of relevant case studies. The patients were tested using panel diagnostics, meaning that they were tested for any mutations in the 49 genes included in the panel only. However, there may have also been variants with a possible link in other genes that were not covered by the testing in the panel. Therefore, a lack of variants found as the result of the panel diagnosis does not mean that there are no other mutations present outside the panel that could be causing the gait anomaly. Because this study is merely taking initial steps to investigate the significance of the genetic variants found in relation to toe walking, our findings do not eliminate the possibility that a variant currently classified as being of uncertain significance could be a cause for the gait anomaly. Our ongoing work indicates the potential to reclassify some variants in the future. For example, many idiopathic toe walkers with a variant of uncertain significance in its heterozygous form display mild symptoms reminiscent of the more severe illnesses experienced by homozygous carriers of the same variant. In addition to having a sample size and the broad scope of genetic testing targets, we could not generate a control group of healthy, normal walking children, as we used retrospective data for ethical reasons. Thus, the existence of variants of relevance in the tested genes in healthy subjects remains unclear. Additionally, the retrospective nature of the data affects the use of validated diagnostic tools to report speech developmental problems and family history. Data on both indicators were collected using our routine method during the anamnestic process, namely through parental statements and examiner observation. For this reason, the aim of this study was purposefully broad—it was an initial attempt to explore the merits of continued research into the possibility of a link between genetic variations and the symptoms.

## Conclusion

We conclude that our findings indicate a sufficient indication for a link between ITW and genetic variants to encourage future research, despite the sample size and the broad scope of our genetic testing targets. Beyond the potential to help us better understand the genetic causes of toe walking, we stress that the panel could be developed further to detect serious conditions early and to clarify treatment implications more quickly. The known genetic disorders that have previously been linked to toe walking are often characterized by a high degree of heterogenicity of symptoms and a variable association between the genotype and the phenotype. We, therefore, think it is likely that our continued research has the potential to identify ITW as a symptom for further conditions, which it was not previously linked to. Despite its limitations, our study shows the merit of continued genetic research into the genetic causes of toe walking. In this way, researchers may eventually be able to move from discussing the possibility of a hereditary cause for some instances of ITW to being able to precisely describe the cause of the condition. Future research may lead to a change in the interpretation of the significance of the variants found in this study.
